# Un hypogonadisme hypogonadotrope congénital combiné révélé par une anomalie de la différentiation sexuelle

**DOI:** 10.11604/pamj.2016.25.134.9882

**Published:** 2016-11-04

**Authors:** Faycal El Guendouz, Ahmed Gaouzi

**Affiliations:** 1 Service d’Endocrinologie, Troisième Hôpital Militaire, Laayoune, Maroc; 2 Département de Pédiatrie Générale, Unité d’Endocrinologie Diabétologie et Maladies Métaboliques, Hôpital d’enfant, Rabat, Maroc

**Keywords:** Hypogonadisme hypothalamohypophysaire congénital, anomalie de la différentiation sexuelle, micro pénis, Congenital hypogonadotropic hypogonadism, abnormality of sex differentiation, micro penis

## Image en médecine

L’hypogonadisme hypothalamohypophysaire congénital (HHC) est défini par une production insuffisante des hormones sexuelles en conséquence d’un dysfonctionnement de la commande hypothalamohypophysaire. On parle de déficit combiné lorsqu’il est associé à une atteinte des autres axes endocriniens. Il est évoqué chez le garçon devant un micropénis, devant un retard pubertaire à l’adolescence et une infertilité à l’âge adulte. Une anomalie de la différentiation sexuelle (DSD) ou une sous virilisation des organes génitaux externe (OGE) est un mode de découverte exceptionnel. Sur les images un enfant issu d’un mariage consanguin dont les parents n’ont consulté qu’à l’âge de 13ans pour une petite taille sévère et une ambiguïté sexuelle de leur enfant. L’interrogatoire a noté l’absence d’anosmie et l’examen clinique a objectivé une taille et un poids inférieurs à moins 4 déviations standards (A). L’examen des OGE a trouvé une DSD classée Prader V, avec un bourgeon de 1 cm, un scrotum d’aspect vulviforme, les gonades intrascrotals de 1 cm (B). Le caryotype était en faveur d’un DSD XY. L’échographie pelvienne et la génitographie ont précisé le type masculin des organes génitaux internes et de l’urètre. Le bilan hormonal était en faveur d’un HHC combiné à un déficit somatrope, les autres axes hypophysaires étaient respectés. L’IRM hypothalamohypophysaire était normale. L’enfant a bénéficié d’un traitement par hormone de croissance recombinante, de 3 cures de micropénis et d’une substitution androgénique retardée jusqu’à un âge osseux de 14ans en vue d’une bonne évolution staturale. L’évolution était très satisfaisante avec un recul de 4ans (A et C).

**Figure 1:**
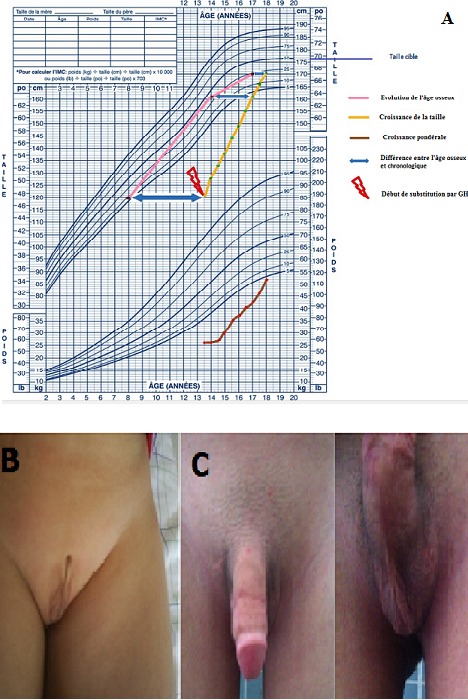
A) courbe de croissance staturo-pondérale illustrant l’évolution sous traitement hormonal; B) OGE avant le traitement; C) OGE après le traitement

